# A Layered NSGA-II Method for LEO Target Census Constellation Design

**DOI:** 10.3390/s26154911

**Published:** 2026-08-04

**Authors:** Changshou Quan, Ping Jian

**Affiliations:** 1 School of Space Information, Space Engineering University, Beijing 101416, China; seustaqcs@163.com; 2 National Key Laboratory of Space Target Awareness, Beijing 101416, China

**Keywords:** LEO target census, constellation design, multi-objective optimization, layered NSGA-II, Starlink

## Abstract

**Highlights:**

**What are the main findings?**

- A layered NSGA-II algorithm is proposed, decomposing the high-dimensional constellation design problem into orbit, configuration, and sensor layers, achieving a hypervolume (HV) of 13.81.
- Compared with classical NSGA-II, MOPSO, MOEA/D, and SPEA2, the proposed method improves convergence speed by approximately 36% while maintaining superior solution diversity.

**What are the implications of the main findings?**

- The layered optimization strategy effectively mitigates the “curse of dimensionality” and local optima trapping in strongly coupled multi-objective space surveillance design.
- The recommended constellation achieves 99.6% coverage with a revisit period as low as 7.6 h and a cost of 0.429, offering an efficient, engineering-ready solution for cataloging mega-constellations like Starlink.

**Abstract:**

To address the pressure of space target census posed by low-orbit mega-constellations such as Starlink, this paper proposes an LEO target census constellation design method based on a layered NSGA-II algorithm. Six decision variables—orbital altitude, inclination, number of orbital planes, number of satellites per plane, sensor field of view, and focal length—are considered. Coverage rate, revisit period, and constellation cost are taken as optimization objectives to establish a multi-objective optimization model. To overcome the issues of slow convergence and susceptibility to local optima in high-dimensional decision spaces faced by classical multi-objective optimization algorithms, the decision variables are divided into the orbit layer, configuration layer, and sensor layer. NSGA-II evolution is performed layer by layer, with elite retention and global archive passing of high-quality solutions. Using high-precision 24-h ephemeris of 496 Starlink satellites generated by STK as the simulation object, the proposed layered NSGA-II is compared with classical NSGA-II, MOPSO, MOEA/D, and SPEA2. Results show that the layered NSGA-II achieves a hypervolume (HV) of 13.16, outperforming the other algorithms. Under the condition of maintaining 99.6% coverage, the recommended constellation solution achieves a revisit period as low as 7.6 h and a constellation cost of 0.429, demonstrating significantly better comprehensive performance than other algorithms. Convergence speed is improved by approximately 36% compared to classical NSGA-II. This method provides an efficient and engineering-applicable optimization approach for LEO target census constellation design.

## 1. Introduction

With the acceleration of space activities, the total number of operational satellites worldwide has reached approximately 25,000, of which 10,689 are Starlink satellites, and the total number of Starlink satellites is expected to reach 42,000 in the future [1]. Meanwhile, space debris is growing exponentially, making near-Earth space increasingly congested and posing a significant challenge to traditional ground-based surveillance. Existing space-based surveillance systems have considerable gaps in coverage timeliness, orbit determination accuracy, and collaborative intelligence, making it difficult to cope with the census pressure brought by LEO mega-constellations such as Starlink. Vigorously developing space-based space surveillance constellations is a future trend.

At present, relatively few studies directly address LEO target census constellation design. Lin et al. [2] systematically analyzed the U.S. Space Tracking and Surveillance System (STSS) currently in orbit from an optical perspective, concluding that this system can track and monitor high-speed moving targets throughout their trajectory, greatly facilitating census and cataloging of LEO objects. Emami et al. [3] proposed a method for space debris monitoring using attitude coordination of multiple spacecraft. Through a coordination algorithm, the spacecraft can perform appropriate maneuvers to point toward the same target. Although the paper does not specifically address constellation design, the attitude maneuvering of multiple spacecraft provides a reference for subsequent constellation design. Considering the large amount of debris in LEO, Du et al. [4] designed three Walker constellations in sun-synchronous orbits, successfully establishing and maintaining a catalog of about 200,000 cm-scale space debris in LEO. To meet the demand for global, full-time coverage in space situational awareness, Hu et al. [5] proposed a space target surveillance constellation design method based on a simulation-comparison approach. After systematically analyzing the orbital distribution characteristics of space targets, they designed a mission mode, key indicators, and constellation constraints for LEO space target census, constructed three basic configurations of space target census constellations, and selected a heterogeneous space target surveillance constellation that satisfies the mission model and capability requirements through simulation comparison. In this paper, “low-orbit target census” refers to the systematic observation and cataloging of resident space objects in low-Earth orbit.

Constellation design involves multiple coupled variables such as orbital altitude, inclination, satellite configuration, and sensor camera parameters. Simultaneously, multiple conflicting objectives such as coverage rate, revisit period, observation arc length, and constellation cost need to be optimized. Essentially, this is a complex high-dimensional multi-objective optimization problem. Traditional multi-objective optimization algorithms generally face the difficulties of search space explosion and slow convergence when dealing with such black-box, multi-constraint, mixed-integer problems. The main reason is the strong coupling effect among the various decision variables of the constellation. Optimizing all variables simultaneously tends to trap the population in a local Pareto front, making it difficult to effectively approach the global optimal solution set. To address these issues, many domestic and international scholars have made various improvements to algorithms. Yan et al. [6] proposed an improved NSGA-II algorithm for remote sensing constellation optimization design, which not only effectively reduces computational complexity but also avoids the problem of target weight selection by ranking the three optimization objectives—coverage percentage, resolution of key observation areas, and number of satellites in the constellation—according to importance. Li et al. [7] from the Beijing Institute of Technology, to solve the high-computational-cost problem in observation satellite constellation design, proposed an NSGA-II algorithm based on a radial basis function (RBF) surrogate model (RBF-NSGA-II). By using a computationally inexpensive RBF surrogate model to approximate the extremely time-consuming constellation simulation model, and applying it to the specific problem of simultaneously optimizing resolution and coverage of observation satellite constellations, they not only reduced computational costs but also improved coverage and resolution, providing decision-makers with multiple feasible Pareto solutions. Dai et al. [8] addressed the optimization design problem of low-orbit regional coverage constellations by proposing an optimization design system for satellite constellations. By analyzing three multi-objective optimization algorithms—NSGA-II, SPEA2, and IBEA—and conducting STK simulations, they demonstrated that all these algorithms can be used to solve regional constellation optimization design problems. To address the uneven latitudinal distribution of coverage resources in low-orbit homogeneous constellations, Jiang et al. [9] proposed an improved constrained-dominance-based non-dominated neighbor immune algorithm (Modified NNIA). Simulation comparisons showed that the improved NNIA algorithm outperforms NSGA-II and MOPSO in both convergence speed and solution diversity, greatly improving constellation design efficiency.

In recent years, layered or decomposition optimization strategies have gained attention in complex engineering design. The core idea is to decompose a high-dimensional coupled problem into several lower-dimensional subproblems, reducing search difficulty through stage-wise evolution. However, the application of such methods in space surveillance constellation design remains unexplored. Several decomposition-based optimization and cooperative coevolution frameworks have been proposed for complex engineering problems. The proposed layered NSGA-II differs from these general frameworks in three aspects:

(1) The decomposition is not arbitrary but physically motivated by the hierarchy of orbit, constellation configuration, and sensor parameters;
(2) Elite solutions from each layer are propagated to the next layer via a global archive, rather than independently optimizing subpopulations;
(3) The hypervolume-based stopping criterion is applied at each layer to balance convergence and diversity. To the best of our knowledge, this specific adaptation for LEO target census constellation design has not been reported.

To this end, this paper proposes a layered NSGA-II algorithm. According to the natural physical hierarchy of surveillance constellation design, the decision variables are divided into the orbit layer (orbital altitude, inclination), configuration layer (number of orbital planes, number of satellites per plane), and sensor layer (field of view, focal length). An outer loop executes standard NSGA-II evolution for each layer sequentially, and high-quality solutions are transferred between layers through an elite retention mechanism and global archive, achieving the unity of dimensionality reduction search and information preservation. This method effectively reduces the variable dimensionality of a single evolution and alleviates the optimization difficulties caused by cross-layer variable coupling. To comprehensively evaluate the performance of the proposed method, using high-precision Starlink target ephemeris derived from the Systems Tool Kit (STK v12.0) as the simulation object, the layered NSGA-II is systematically compared with classical NSGA-II, MOPSO, MOEA/D, and SPEA2 under a unified experimental framework, thereby verifying the superiority of the layered optimization strategy in strongly coupled multi-objective constellation design.

## 2. Problem Modeling for Constellation Design

### 2.1. Surveillance Constellation Configuration Parameters

Constellation configuration is generally determined by the six orbital elements of satellites, the number of orbital planes, and the number of satellites per orbital plane. Taking the Walker constellation as an example, it uniquely determines the global seamless coverage satellite distribution using the three parameters “T/P/F” (total number of satellites/number of orbital planes/phase factor) [10]. In a Walker constellation, each satellite has the same orbital altitude, inclination, and eccentricity, ensuring that all satellites in the constellation have the same evolution characteristics when facing disturbances such as atmospheric drag, Earth’s gravitational perturbations, and tides, thus guaranteeing the stability of constellation performance.

**Figure 1:**
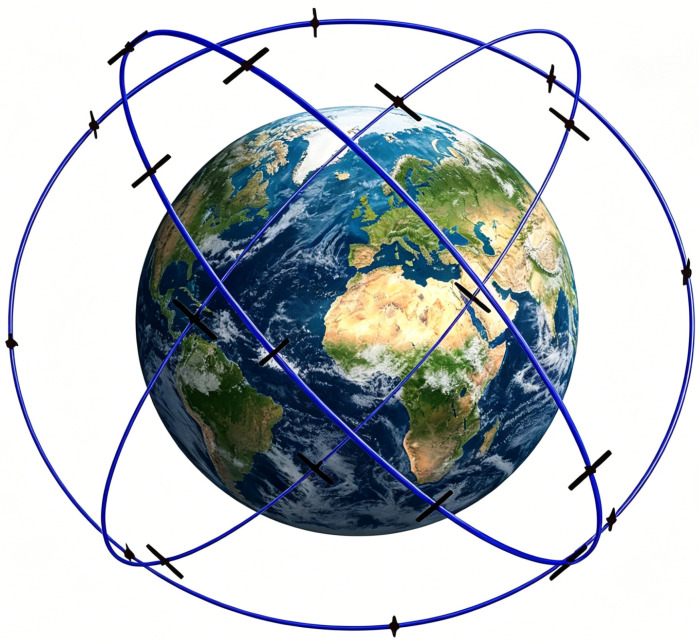
Walker constellation schematic.

As introduced above, a Walker constellation is a circular orbit constellation consisting of T uniformly distributed orbital planes, with P uniformly distributed satellites in each plane. Satellites in each orbital plane of a Walker constellation have the same right ascension of the ascending node (RAAN), and all satellites share the same orbital altitude and inclination. A schematic diagram of the Walker constellation is shown below (Figure 1).

Since the orbital planes in the constellation are uniformly distributed around the Earth, the RAAN of satellites in each orbital plane is the same. Let the number of orbital planes in the constellation be P, and the minimum RAAN be ${RAAN}_{1}$, then the RAAN of satellites in orbital plane i, ${RAAN}_{i}$, can be expressed as

(1) $${RAAN}_{i}={RAAN}_{1}+\frac{360}{P}(i-1),\;i=1,2,\;...\;,\;P$$

Let the phase factor be F, 0≤F<P. Let the number of orbital planes be P, and the number of satellites per orbital plane be T. Then, the true anomaly v of the j-th satellite in orbital plane i can be expressed as

(2) $$v=\frac{360}{T*P}*F*(i-1)+\frac{360}{T}(j-1),\;i=1,2,...\;,P;\;j=1,2,...\;,\;T$$

Thus, setting a reference RAAN allows the determination of RAANs for all satellites in the constellation based on the Walker configuration parameters T, P, F. Similarly, the true anomalies can be determined. Therefore, determining a Walker constellation requires only six parameters: orbital altitude, inclination, RAAN, number of orbital planes, number of satellites per orbital plane, and phase factor. Since the constellation will be used long-term, the RAAN and phase factor have little impact on constellation performance for long-term observation. Thus, constellation configuration selection requires choosing four parameters: orbital altitude, inclination, number of orbital planes, and number of satellites per orbital plane. These four parameters uniquely determine a constellation.

### 2.2. Sensor Model

During the optical observation of Starlink targets by a surveillance constellation, the optical sensor on each surveillance satellite is the core payload for target acquisition. Constrained by the strict limitations on payload volume, mass, and power consumption of microsatellite platforms, space-based space target optical surveillance systems employ various trade-offs: Canada’s NEOSSat satellite features a 15 cm aperture Maksutov telescope with an F/5.88 focal ratio and a field of view of approximately 0.85°, equipped with an E2V 1024 × 1024 CCD, capable of detecting 20 Mv targets [11]; the Sapphire satellite achieves high-sensitivity dark target observation through an efficient low-noise CCD combined with a high-throughput optical system [12]; and domestic researchers have designed an 8° × 8° wide-field optical camera weighing only 5 kg with detection capability exceeding 13 Mv, validating the potential of wide-field compact optical–mechanical systems for LEO target census applications [13]. The US SBV sensor, as a first-generation space-based space surveillance payload, operates in the 450–950 nm band using a passive optical detection system. However, due to its limited field of view (approximately 60 μrad/pixel), it is mainly aimed at GEO belt census and has limited search coverage capability for fast-moving LEO targets. The mission experience and design parameters mentioned above provide engineering justification for the selection of sensor parameter ranges in this paper.

Assuming an ideal optical system, the detection capability is determined by two core parameters: the field of view (FOV) $\theta_{FOV}$ and the focal length f. $\theta_{FOV}$ defines the spatial angular range of a single image, while focal length f, together with the detector pixel size, determines the instantaneous field of view (IFOV), affecting target resolution capability and detection probability. Based on the design experience of existing space surveillance payloads [11,12,13], $\theta_{FOV}$ is typically valued between 0.1 and 0.5 rad (approximately 5.7° to 28.6°), and focal length between 0.3 and 1.0 m. This parameter range is chosen to cover most of the low-Earth orbit space, achieving a comprehensive trade-off between census and tracking of Starlink targets.

At any time t, whether a surveillance satellite can effectively detect a Starlink target depends on three visibility constraints:

- Earth occultation constraint

The target must be above the surveillance satellite’s horizon, i.e., the closest distance d between the target-satellite vector $r_{T}-r_{S}$ and the Earth’s center must be greater than the Earth’s radius $R_{E}$, calculated using the line segment–sphere center shortest distance method.

- Solar phase angle constraint

To avoid detector saturation or an insufficient signal-to-noise ratio, the angle ψ between the target-satellite vector and the target-sun vector must not exceed the maximum allowable phase angle $\psi_{max}$. Since the optical detectability of LEO targets is highly dependent on the trade-off between phase angle illumination conditions and exposure time [14], referring to the design experience in [11,12], this paper sets $\psi_{max}=50{}^{\circ}$.

- Minimum elevation angle constraint

To overcome atmospheric attenuation and Earth background radiation (mainly Earth limb stray light in space-based detection), the elevation angle ε of the target in the surveillance satellite’s local horizontal coordinate system must not be less than the minimum working elevation angle $\varepsilon_{min}=10{}^{\circ}$, consistent with the typical design value of the SBV sensor.

Formally, the visibility function can be expressed as

(3) $$v_{i,j}(t)=\left\{\begin{array}{c} 1,\;d(t)>R_{E}\wedge \psi (t)\leq \psi_{max}\wedge \varepsilon (t)\geq \varepsilon_{min}\\ 0,\;else \end{array}\right.$$

where i represents the i-th surveillance satellite, and j represents the j-th Starlink target.

The optical performance of the sensor is not directly reflected in the above visibility constraints but affects system performance mainly through the following two pathways: a wider field of view $\theta_{FOV}$ covers a larger space area, significantly improving search coverage efficiency for Starlink target groups [13,14]; an appropriately long focal length f improves angular resolution, facilitating target confirmation or high-precision orbit determination at greater distances, but simultaneously reduces the effective coverage range and extends the revisit time [15]. Therefore, $\theta_{FOV}$ and f constitute a pair of mutually constraining design parameters. Within the multi-objective optimization framework, sensor parameters and orbit configuration parameters are co-optimized to achieve a balance among coverage, revisit period, and constellation cost while meeting minimum constraint conditions.

We emphasize that the visibility model adopted in this work is a simplified geometric proxy. The condition for a target to be considered “visible” is given by Equation (3), which only considers Earth occultation, solar phase angle (<50°), and minimum elevation angle (>10°). The sensor design variables (FOV and focal length) are not used in the visibility calculation in this simplified model. Instead, FOV is treated as a design variable for future extension to a physical detection model, and focal length is retained for consistency but has no effect on the current objective functions.

Thus, the reported performance metrics (coverage, revisit time, observation arc length) are based solely on geometric visibility and should not be interpreted as actual optical detection probabilities. A realistic optical model would require parameters such as aperture, target brightness, SNR, and integration time. For space-based surveillance in LEO, high-fidelity models have been developed in [16] and [17]. Incorporating such models would significantly increase the computational cost of each function evaluation. In this paper, we intentionally use the geometric proxy to keep the optimization tractable, and we defer the integration of physical detection models to future work.

The geometric visibility conditions (Equation (3)) are necessary but not sufficient. A target must also produce a sufficiently high signal-to-noise ratio (SNR) at the sensor. Following standard radiometric models [16,17], the SNR for a point target can be approximated as

(4) $$SNR=\frac{\Phi_{target}\cdot A_{ap}\cdot \tau_{opt}\cdot QE\cdot t_{exp}}{\sqrt{N_{photon}^{2}+N_{dark}^{2}+N_{read}^{2}}}$$

where $A_{ap}=\pi (D/2{)}^{2}$ is the aperture area (determined by the effective aperture diameter D), $\tau_{opt}$ is the optical transmittance, QE the quantum efficiency, $t_{exp}$ the exposure time, and the denominator represents the total noise. For a distant target, the irradiance $\Phi_{target}$ obeys $\Phi_{target}\propto 1/R^{2}$, with R being the distance.

The field of view (FOV) and focal length f affect the detection capability because the FOV determines the angular coverage and the collected background light, while f together with the detector pixel size sets the instantaneous FOV and thus the angular resolution. A detailed derivation shows that the maximum detection range $R_{max}$ for a given SNR threshold ${SNR}_{min}$ can be expressed as [18]

(5) $$R_{max}\propto \sqrt{\frac{D^{2}\cdot QE\cdot t_{exp}}{{SNR}_{min}\cdot FOV}}$$

and the FOV is related to f by $FOV\approx (N_{pix}\cdot p)/f$ for a detector with pixel size p and $N_{pix}$ pixels. Hence, both FOV and focal length directly influence $R_{max}$, and therefore affect coverage, revisit time, and observation arc length.

In the present implementation, we retain the geometric visibility model (Equation (3)) for computational efficiency, as a full optical model would increase the evaluation cost by orders of magnitude. Nevertheless, the above relations demonstrate that the sensor design variables are physically meaningful and can be integrated into the layered optimization framework in future work without structural changes.

### 2.3. Optimization Metric Model Construction

According to the optimization metrics to be considered for LEO target census tasks, four metrics are selected: coverage rate, revisit period, observation arc length, and constellation cost. These are used as inputs to construct the surveillance constellation optimization metric model, enabling quantitative evaluation of the surveillance constellation and laying the foundation for subsequent constellation design optimization.

- Coverage Rate

Coverage rate is the ratio of the number of targets covered by the constellation to the total number of targets within a given observation period, effectively reflecting the constellation’s utilization efficiency. Let M be the total number of targets, and m be the number of targets covered by the constellation. Then, the coverage rate can be expressed as

(6) $$f_{1}=\frac{m}{M}$$

- Revisit Period

The revisit period is the time interval between two consecutive starts of access to a target by the constellation. For convenience in subsequent metric optimization, the average revisit time is taken, i.e., the average of time intervals for all targets accessed two or more times. This metric reflects the constellation’s comprehensive revisit capability for all targets, providing an overall measure of the constellation’s timeliness in census tasks. A smaller average revisit time indicates better constellation coverage capability and a lower probability of target loss. Let $M_{tar}$ be the number of targets accessed two or more times, N be the total number of accesses for target m (which can be accessed two or more times), and ${ts}_{m}^{n}$ be the n-th access start time for target m. Then, the average revisit time can be expressed as

(7) $$f_{2}=\frac{1}{M_{tar}}\sum_{m=1}^{M_{tar}}\sum_{n=1}^{N}{(ts}_{m}^{n+1}-{ts}_{m}^{n})$$

- Constellation Cost

Constellation cost includes the production cost and launch cost. Production cost is determined by the satellites themselves, the payloads they carry, and the number of satellites. Launch cost is determined by the altitude and inclination of the orbital plane of each satellite in the constellation, the number of satellites per orbital plane, and the number of orbital planes. To eliminate the effects of scale and magnitude for the convenient comparison of metrics, this paper uses the relative cost for quantitative calculation, expressed as follows:

(8) $${\;f}_{3}=\omega_{nums}\times \frac{num\;s_{min}}{nums}+\omega_{nump}\times \frac{num\;p_{min}\;}{nump}+\omega_{h}\times \frac{h_{min}}{h}+\omega_{i}\times \frac{i_{min}}{i}$$

where nums represents the number of satellites per orbital plane, nump represents the number of orbital planes, h represents orbital altitude, i represents orbital inclination, and $\omega_{nums}$, $\omega_{nump}$, $\omega_{h}$, and $\omega_{i}$ represent their respective weights, which sum to 1.

In summary, for ease of solution, the objective function is converted to a cost type, seeking its minimum. The optimization objective function composed of surveillance constellation optimization metrics is

(9) $$\left\{\begin{array}{c} min\;F(x)=(-f_{1}(x),{\;f}_{2}(x),-f_{3}(x))\\ subject\;to\;x\in \Omega \end{array}\right.$$

### 2.4. Use of GenAI

During the preparation of this study, the authors used Deepseek (version: Deepseek-V3) as an auxiliary tool for data collection and data analysis, specifically:

1. Data Collection: Deepseek was used as an information retrieval aid to locate publicly available TLE data sources for Starlink satellites. The AI tool provided a link to the Space Mapper website (https://spacemapper.cn/) (accessed on 14 April 2026), from which the authors manually downloaded the required TLE orbital data for 496 Starlink satellites. The authors independently verified the data source and downloaded all data—no automated data scraping or uploading of raw data to the AI was performed.

2. Data analysis: Deepseek was used as a coding assistant to help write MATLAB R2020b scripts for processing the STK-generated ephemeris data. This included scripts for reading the raw .e files, interpolating data onto a unified time vector. The authors provided detailed prompts to the AI, manually reviewed, validated, and adjusted all generated code, and take full responsibility for the correctness of the data processing and analysis.

The authors take full responsibility for the integrity and correctness of all data processing and analysis.

## 3. NSGA-II Algorithm Based on Layered Optimization Framework

As mentioned earlier, the sensor chooses the field of view and aperture area as decision variables, while the constellation configuration chooses orbital altitude, inclination, number of orbital planes, and number of satellites per orbital plane as decision variables. Facing such a multi-objective optimization problem with multiple optimization metrics and multiple decision variables, conventional geometric analytical methods cannot yield accurate analytical solutions. To address this issue, this paper proposes an NSGA-II algorithm based on a layered optimization strategy. By layering the decision variables such that each layer has no more than three variables, it can somewhat alleviate the problem that Pareto dominance is only suitable for multi-objective optimization problems with fewer than three objectives.

### 3.1. Basic Idea of the Algorithm

In constellation optimization problems, the six decision variables have a natural hierarchical structure: orbital altitude h and inclination i determine the fundamental operating laws of satellites; the number of orbital planes $N_{p}$ and the number of satellites per plane $N_{s}$ determine the spatial distribution density; and sensor field of view ${\;\theta }_{FOV\;}$ and focal length f fine-tune the final coverage quality and positioning accuracy. Coupling between these layers is relatively weak, and thus a “framework-first, details-later” search strategy can be adopted.

The core idea of layered NSGA-II is to divide all variables into L=3 physical layers. The l-th layer activates only its own variables, while variables from upper layers remain frozen. A standard NSGA-II algorithm runs independently in each layer to obtain the Pareto optimal solution set for that layer under the currently frozen variables. Subsequently, through an elite retention mechanism, excellent individuals from the current layer are written back to the global population, replacing the worst individuals, and the variables of the current layer are frozen before proceeding to the next layer. The outer loop repeatedly traverses all layers until the change in the hypervolume indicator falls below a threshold $\eta_{HV}$.

The essence of this strategy is to decompose a full-space optimization with M objectives and d-dimensional variables into several sub-optimizations, each with M objectives and ${\;d}_{l}$ dimensions ($d_{l}\leq 2$). In each layer cycle, the dimensionality of the objective space remains unchanged, but the decision space dimensionality is significantly reduced. Therefore, the selection pressure of NSGA-II can be maintained at a high level, effectively avoiding the issues of crowding distance failure and population stagnation in high-dimensional decision spaces.

However, a naive hierarchical optimization suffers from the risk of one-way information flow: an optimal solution selected in an upper layer may perform poorly in a lower layer, yet there is no mechanism to revise the upper layer’s choice. To address this, an elite migration mechanism is designed to achieve bidirectional flow of high-quality solutions between layers.

Let the non-dominated solution set obtained after optimizing layer l be $P_{l}^{*}$. From it, the top $\pi_{l}\cdot N_{l}$ elite individuals are selected based on non-domination rank and crowding distance ($\pi_{l}$ is the elite write-back ratio). These individuals replace the worst individuals in the global population G:

(10) G←(G\W)∪ε

where W is the set of inferior individuals in the global population (those with the smallest crowding distance and highest non-domination rank), and ε is the set of elite solutions selected from the current layer. After the replacement, the variables of the current layer are frozen, and optimization of the next layer proceeds on this basis. This mechanism ensures bidirectional information flow and global coordination between layers, avoiding information isolation that may result from simple layering.

Furthermore, classic NSGA-II typically uses a fixed number of generations as a termination condition. However, the required evolutionary generations vary greatly for problems of different scales; a fixed generation number either incurs redundant computation or leads to insufficient evolution. In this paper, the hypervolume (HV) is adopted as an adaptive convergence criterion for the outer loop. HV simultaneously reflects both the convergence and diversity of a solution set, and is defined as

(11) $$HV(F)=\lambda (\cup_{f\in F}\left[f,r\right])$$

where F is the non-dominated front of the current population, and [f,r] denotes the M-dimensional hypercube region with the single Pareto solution f and the reference point $r=(r_{1},...,r_{M})$ as opposite vertices. Since there are three objectives, M=3. λ is the volume measure of the M-dimensional hypercube.

After each complete round of three-layer optimization, the hypervolume value ${HV}_{cur}$ of the current global population is computed and compared with the hypervolume value ${HV}_{prev}$ from the previous round. If,

(12) $$\frac{\left|{HV}_{cur}-{HV}_{prev}\right|}{{HV}_{prev}}<\varepsilon_{HV}$$

the algorithm is considered to have converged, and the outer loop terminates automatically. This strategy adaptively controls the computational cost while ensuring solution quality, making the algorithm more adaptable to constellation design problems of different scales.

### 3.2. Algorithm Framework and Process

**Figure 2:**
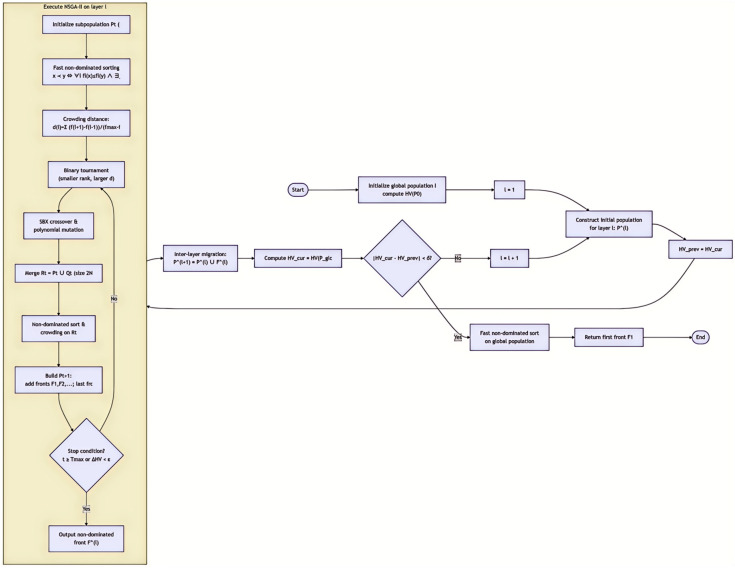
Flowchart of layered NSGA-II algorithm.

The flowchart of layered NSGA-II is shown in Figure 2, and the corresponding pseudocode is presented in Algorithm 1.
**Algorithm 1.** Layered NSGA-II Algorithm1:Initialize population P with size $N_{l}$ for each layer2:Initialize archive A[l]=∅ for l=1 to L3:Initialize frozen[l]=∅ for l=1 to L4:Set ${HV}_{prev}=10^{-5}$
5:
6:repeat7:        for l=1 to L do8:        //Activate current layer variables, freeze upper layer variables9:        
$P_{l}=ExtractActivateVariables(p,P_{set}[l],frozen[l-1])$
10:
11:        if l=1 then12:          
$P_{l}^{0}=P_{l}$
13:        else14:          
$P_{l}^{0}=BuildLayerPopulation(A[l-1],N_{l},P_{set}[l])$
16:        end if17:
18:        for g=1 to $G_{l}$ do19:          
$Evaluate(P_{l}^{g-1},S_{l})$
20:          
$F=FastNonDominatedSort(P_{l}^{g-1})$
21:          
ComputeCrowdingDistance(F)
22:          
$Q=GeneticOperators(TournamentSelect(P_{l}^{g-1},F),p_{c},p_{m})$
23:          
$R=P_{l}^{g-1}\cup Q$
24:          
$P_{l}^{g}=EnvironmentalSelection(R,N_{l})$
25:        end for26:        //Elite migration and variable freezing27:        
$E_{l}=SelectTopPercent(P_{l}^{g-1},\pi_{l})$
28:        
$A[l]=UpdateArchive(A[l],E_{l})$
29:        
$frozen[l]=frozen[l]\cup P_{set}[l]$
30:        
$P=WriteBack(P,E_{l},P_{set}[l])$
31:end for32://Global convergence check33:${HV}_{cur}=ComputeHypervolume(P)$34:Until $\frac{\left|{HV}_{cur}-{HV}_{prev}\right|}{{HV}_{prev}}<\varepsilon_{HV}$
35:${HV}_{prev}={HV}_{cur}$36://Output Pareto front37:F=FastNonDominatedSort(P)38:return F[1]


Within each layer, the standard NSGA-II implementation is used: fast non-dominated sorting, crowding distance calculation, SBX crossover, and polynomial mutation. Due to the low dimensionality of intra-layer variables and constant objective dimensionality, NSGA-II can quickly converge to the layer’s Pareto front while maintaining population diversity.

The frozen variables for a given layer are initialized using non-dominated solutions from the previous layer’s Pareto front. Specifically, after optimizing the orbit layer, the resulting set of Pareto-optimal orbit parameters (e.g., altitude, inclination) are stored in a global archive. For the configuration layer, each individual picks one of these orbit solutions as frozen variables. This ensures that lower-layer optimization starts from promising upper-layer choices.

In the current implementation, decisions made in an upper layer are frozen and not revised by lower-layer optimization. This is a limitation of the hierarchical approach. However, the global archive maintains a set of diverse elite solutions from all layers; when the next outer cycle starts, new upper-layer individuals can be seeded from the archive, indirectly allowing some feedback. A direct feedback mechanism is left for future work.

Integer variables (e.g., number of satellites per plane, number of planes) are handled as follows: during initialization and variation (SBX crossover and polynomial mutation), they are treated as real numbers; before evaluation, they are rounded to the nearest integer. The same rounding is applied to any integer variable that appears in the objective functions or constraints.

The global archive stores all non-dominated solutions found across all layers. It is updated after each generation using Pareto dominance: any newly generated solution that is not dominated by any existing archive solution is added; any archive solution that becomes dominated by the new solution is removed. To prevent unbounded growth, the archive size is limited to 200. When the limit is exceeded, crowding distance (as in NSGA-II) is used to remove solutions with the smallest crowding distance, preserving diversity.

### 3.3. Comparison with Classic NSGA-II

Table 1 lists the main differences between layered NSGA-II and classical NSGA-II.

## 4. Simulation Analysis of Constellation Design

This chapter describes the comparative simulation experiments for constellation design. First, the compared algorithms and their parameter settings are briefly introduced. Then, the simulation scenario and the generation of STK ephemeris data are described. Finally, the convergence performance and solution quality are systematically compared.

### 4.1. Comparison Algorithms and Parameter Settings

To validate the effectiveness of the proposed layered NSGA-II algorithm, the following four typical multi-objective evolutionary algorithms are selected as baselines: classical NSGA-II, Multi-Objective Particle Swarm Optimization (MOPSO), Multi-Objective Evolutionary Algorithm based on Decomposition (MOEA/D), and Strength Pareto Evolutionary Algorithm 2 (SPEA2). All algorithms use the same high-fidelity objective function and STK ephemeris data, and the total number of evaluations is kept approximately consistent to ensure fair comparison.

#### 4.1.1. Classic NSGA-II

Classical NSGA-II optimizes all six decision variables directly without introducing a layering strategy: population size N=100, number of generations G=150, crossover probability $p_{c}=0.9$, mutation probability $p_{m}=0.1$, SBX crossover distribution index $\eta_{c}=20$, polynomial mutation distribution index $\eta_{m}=20$. Each generation includes non-dominated sorting, crowding distance calculation, and elite environmental selection.

#### 4.1.2. Multi-Objective Particle Swarm Optimization (MOPSO)

MOPSO uses an external archive to maintain non-dominated solutions, with archive capacity $N_{rep}=100$, and the number of particles equal to the population size. Inertia weight ω=0.5, decaying with $\omega_{damp}=0.9$; individual learning factor $c_{1}=1.0$; and social learning factor $c_{2}=2.0$. To enhance exploration capability, a mutation probability of 0.1 and mutation scale of 0.1 are set. Leader selection is based on the crowding distance roulette wheel strategy.

#### 4.1.3. Multi-Objective Evolutionary Algorithm Based on Decomposition (MOEA/D)

MOEA/D uses the Tchebycheff decomposition method. The number of weight vectors equals the population size N=100 (generated by the simplex lattice method). In each generation, neighborhood size T=20. Crossover and mutation parameters are the same as those in NSGA-II. The ideal point z* is updated dynamically during evolution.

#### 4.1.4. Strength Pareto Evolutionary Algorithm 2 (SPEA2)

The population size and external archive size for SPEA2 are both set to 100. k-nearest neighbor density estimation is used, with $k=\sqrt{N+N_{archive}}$. Fitness assignment is based on the sum of strength values and density values. In environmental selection, if the number of non-dominated solutions exceeds the archive capacity, the most densely populated individuals are removed through iterative truncation. Mating selection uses a binary tournament, and crossover and mutation parameters are consistent with those mentioned earlier.

#### 4.1.5. Layered NSGA-II

Layered NSGA-II divides the six decision variables into three layers: orbit layer (altitude, inclination), configuration layer (number of orbital planes, number of satellites per plane), and sensor layer (field of view, focal length). Each layer has a population size N=100, intra-layer generations G=50, and elite write-back ratio π=0.5. The outer loop executes a maximum of 50 times, with convergence threshold $\eta_{HV}=10^{-5}$. The remaining genetic operator parameters are consistent with classical NSGA-II.

To quantitatively compare the quality of the Pareto fronts obtained by different algorithms, hypervolume (HV) is introduced as an evaluation metric. The reference point is set to r=(0.1, 24.1, 1.1, 1.1). A larger HV value indicates that the solution set is closer to the reference point in the objective space and has a wider distribution. All HV values are computed using the same reference point and compared against other algorithms under the same number of evaluations. In addition, scatter plots of the final populations in the three-dimensional objective space (coverage—revisit time—cost) are presented for each algorithm, and a recommended solution selected by the weighted sum method is given, with specific parameters for engineering reference.

### 4.2. Simulation Scenario Settings

This paper takes the currently in-orbit Starlink low-orbit mega-constellation as the surveillance target, aiming to design a dedicated optical surveillance constellation for efficient census and cataloging of Starlink targets. The target constellation consists of 496 low-orbit satellites with orbital altitudes distributed between 140 km and 550 km, and inclinations generally around 53°, characterized by large numbers, concentrated distribution, and high-speed motion.

To obtain high-fidelity target position information, this paper uses STK’s high-precision orbit propagation model to generate Starlink satellite ephemeris data. The specific steps are to establish all Starlink targets in STK based on publicly available TLEs, set the simulation period to 24 h starting from 00:00:00 UTC on 14 April 2026, and output the position vector (in km) of each satellite in the Earth-centered inertial coordinate system at 60-s intervals. The exported ephemeris files are uniformly read by MATLAB and interpolated onto the same time vector, forming a $3\times N_{S}\times N_{t}$ position matrix serving as a static target library for subsequent objective function evaluations.

The performance of the surveillance constellation is guaranteed by three typical visibility constraint conditions: ① Earth occultation constraint: the distance between the target-satellite line of sight and the Earth’s center must be greater than the Earth’s radius; ② Solar phase angle constraint: the Sun-target-satellite angle must not exceed 50° to avoid detector saturation or insufficient signal-to-clutter ratio; ③ Minimum elevation angle constraint: the elevation angle of the target in the surveillance satellite’s body coordinate system must not be less than 10°. These constraints are uniformly quantified as indicator functions of time and embedded in the objective function calculation.

### 4.3. Convergence Curve

**Figure 3:**
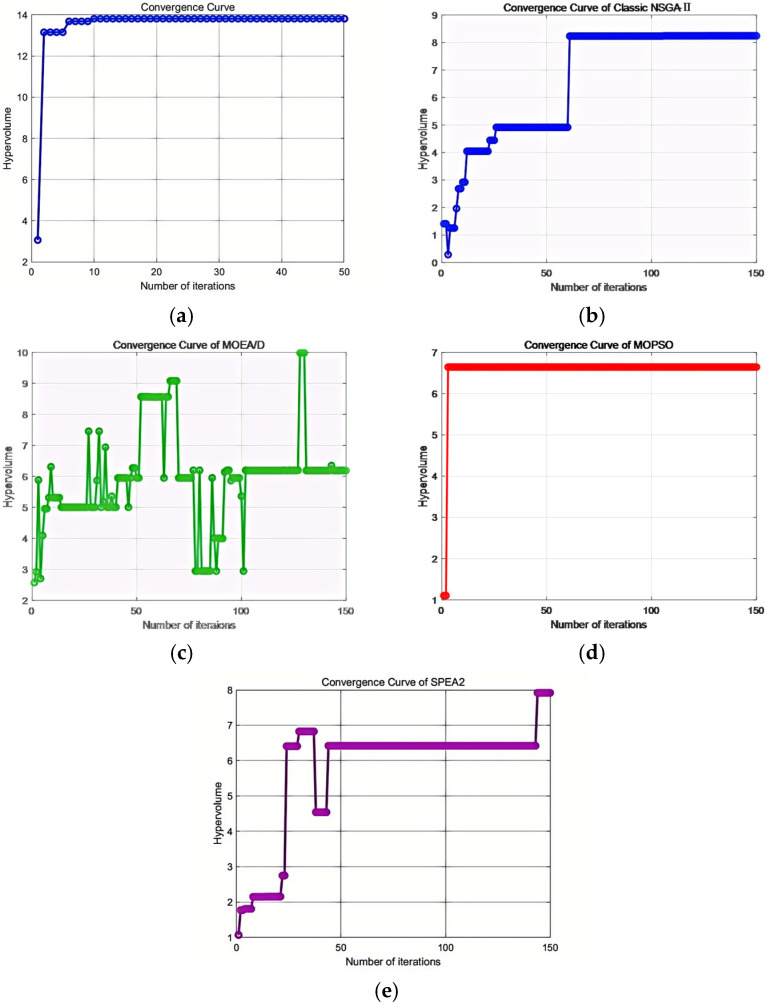
Convergence curves of different algorithms: (**a**) Layered NSGA-II, (**b**) Classical NSGA-II, (**c**) MOPSO, (**d**) MOEA/D, (**e**) SPEA2. Note: The x-axis shows the number of iterations. For all algorithms, each generation evaluates exactly 100 offspring individuals (the population size). Therefore, the total number of function evaluations (FEs) equals the (number of generations + 1) × 100, counting the initial population. The layered NSGA-II uses 3 layers × 50 generations per outer cycle, corresponding to 15,000 FEs per cycle, but terminates earlier due to the HV convergence criterion.

Figure 3 shows the hypervolume (HV) convergence curves for each algorithm when the total number of evaluations is $1.6\times 10^{4}$. The horizontal axis represents the actual number of objective function iterations, and the vertical axis represents the HV value calculated with the reference point r=(0.1, 24.1, 1.1).

As can be seen from the figures, layered NSGA-II exhibits a rapid HV growth trend in the early stage, reaching a convergence plateau much earlier than the other algorithms, with a final HV value converging to about 13.81, outperforming classical NSGA-II (approx. 8.25), MOEA/D (approx. 6.19), MOPSO (approx. 6.64), and SPEA2 (approx. 7.92). In terms of convergence speed, the number of function evaluations required to reach 90% of its final HV is about 36% less than that of classical NSGA-II, which is approximately 36% less than classical NSGA-II, further illustrating that the layering strategy can effectively accelerate the search process.

### 4.4. Performance Comparison of Recommended Solutions

**Figure 4:**
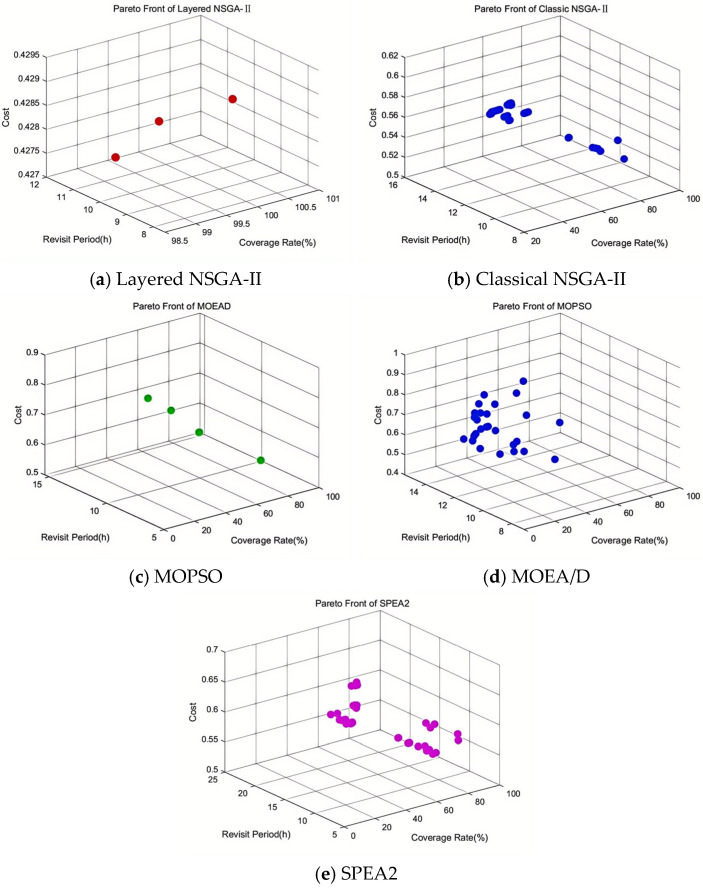
Three-dimensional approximate Pareto fronts of different algorithms.

Figure 4 shows the approximate Pareto front distributions of the algorithms in the three-dimensional objective space (coverage rate $f_{1\;}$, revisit time $f_{2}$, cost $f_{3}$).

It is evident from the figures that compared to the other four algorithms, although the number of frontier solutions for layered NSGA-II is relatively smaller, under the same coverage rate, the quality of the revisit period and cost metrics is significantly higher. Furthermore, classic NSGA-II and MOPSO show fronts concentrated in certain regions, leading to a significantly lower solution quality.

For ease of engineering decision-making, a recommended solution is selected from the Pareto front of each algorithm using the weighted sum method with weight vector (0.4, 0.25, 0.35). Table 2 lists the specific parameters and objective values of three recommended solutions for each algorithm.

### 4.5. Experimental Conclusions

1. The advantages of layered NSGA-II in convergence speed and front quality mainly stem from the following two points:

(1) Effective dimensionality reduction in the search space. Each evolution activates only two variables of the current layer, allowing the selection pressure of NSGA-II to be well maintained. The crowding distance between individuals can be effectively measured, helping to maintain population diversity and continuously approach the Pareto front.

(2) The inter-layer elite write-back mechanism. By replacing the worst individuals in the global population with the frontier elites from the current layer, high-quality solutions are transferred across layers, avoiding overall performance degradation due to stagnation in a single layer.

2. Analysis of performance differences with other algorithms:

(1) Comparison with MOEA/D: MOEA/D relies on the uniformity of weight vectors. The four-objective weight generation algorithm struggles to ensure boundary coverage when the population size is 100. After the layering strategy decomposes the decision space, each layer still has a four-objective optimization problem, but the reduced variable dimensionality makes it easier for the population to explore extreme solutions.

(2) Comparison with MOPSO: MOPSO converges relatively quickly, but external archive maintenance and density estimation in a high-dimensional objective space can lead to uneven front distribution. Layered NSGA-II adjusts variables layer by layer, reducing the complexity of the objective space and thus obtaining a more uniform Pareto front.

(3) Comparison with SPEA2: SPEA2’s refined fitness assignment and truncation operations are effective in low-dimensional decision spaces, but computational overhead is high in full-variable optimization, and density estimation is sensitive to the parameter k. Layered NSGA-II avoids generating overly dense regions through layer-by-layer evolution, and the global elite write-back mechanism controls archive maintenance overhead.

## 5. Conclusions

Aiming at the surveillance constellation design problem for Starlink LEO target census, this paper proposes a layered NSGA-II algorithm. By dividing orbit, configuration, and sensor parameters into three layers and optimizing them layer by layer, the algorithm effectively mitigates the curse of dimensionality faced by traditional multi-objective evolutionary algorithms when solving multi-objective mixed-integer optimization problems. In simulation scenarios based on STK high-precision ephemeris, comparative experiments with classical NSGA-II, MOPSO, MOEA/D, and SPEA2 demonstrate the following:

(1) The hypervolume (HV) indicator of layered NSGA-II is significantly better than that of other algorithms, with HV improvements of approximately 26.8% to 52.6%.
(2) The Pareto front obtained by the algorithm is wider and more uniformly distributed, providing a richer set of optimal constellation design choices.
(3) The recommended constellation scheme selected based on the weighted sum method achieves a better comprehensive performance in terms of coverage rate, revisit time, and cost, validating the engineering application value of the layered optimization strategy.

The constellation design in this work is based on deterministic target ephemerides and ideal visibility conditions. In real LEO target census missions, uncertainties in initial orbit determination, sensor measurements, and orbit propagation nonlinearities can significantly affect the actual coverage performance. Several methods have been proposed to handle such uncertainties, including nonlinear semi-analytic trajectory estimation [19], time-varying directional state transition tensors [20], and differential algebra-based nonlinear filtering [21]. Although the current optimization framework does not explicitly account for these uncertainties, it can be extended by incorporating uncertainty propagation into the coverage evaluation step. For example, one could evaluate the expected coverage over a distribution of initial orbit errors or use robust optimization techniques. We consider this an important direction for future work.

It should be noted that the orbital altitude, number of orbital planes, sensor field of view, and revisit time are inherently coupled. In the proposed layered optimization, decisions from the upper layer (orbit) are frozen when optimizing the lower layers (configuration and sensor), which may prevent the algorithm from reaching the global optimum if strong coupling exists. However, the elite retention mechanism and the global archive alleviate this issue by preserving diverse high-quality solutions across layers. Future work could incorporate a feedback loop that allows lower-layer results to adjust upper-layer decisions. Furthermore, future research will explore dynamic layer scheduling and online mission planning methods to further enhance the adaptive capability of surveillance constellations.

## Figures and Tables

**Table 1:** Main differences between layered NSGA-II and classical NSGA-II.

Aspect	Classic NSGA-II	Layered NSGA-II
Search Space	Simultaneous optimization of all variables	Divided into layers, each optimized independently
Elite Retention	Global non-dominated sorting + crowding distance	Intra-layer elite retention + global write-back
Handling of Variable Coupling	Prone to becoming stuck in local Pareto fronts	Mitigates coupling through layer-by-layer freezing
Convergence Speed	Slow in high dimensions	Faster achievement of hypervolume threshold
Generality	Applicable to any MOP without prior knowledge	Relies on physical layering, but applicable to multiple constellation design types

**Table 2:** Specific parameters and objective values of three recommend solutions for each algorithm.

Parameter/Metric	Layered NSGA-II	Classic NSGA-II	MOPSO	MOEA/D	SPEA2
Altitude (km)	1000	961.36	626.2	986.5	986.9
	988.41	979.15	656.8	998.1	996.5
	1000	982.73	987.6	1000	988.2
Inclination (°)	95.8	97.3	79.3	98.0	66.3
	98.0	97.9	64.7	98.0	97.4
	98.0	97.4	90.4	98.0	98.0
Number of planes $N_{p}$	4	3	2	3	3
	4	3	3	3	3
	4	3	4	3	3
Satellites per plane $N_{s}$	6	6	5	6	6
	6	6	4	6	6
	6	6	5	6	6
FOV (rad)	0.50	0.50	0.42	0.50	0.50
	0.50	0.43	0.38	0.50	0.27
	0.40	0.50	0.43	0.49	0.36
Focal length (m)	0.99	0.85	0.53	0.61	0.64
	1.00	0.85	0.57	0.43	0.45
	0.83	0.85	0.39	0.49	0.40
Coverage rate (%)	99.6	99.2	52.8	99.4	99.6
	99.6	99.0	53.4	99.6	98.6
	99.6	99.6	99.6	99.4	99.0
Avg. revisit period (h)	7.6	11.6	13.4	8.5	15.8
	10.3	13.2	15.0	15.4	15.8
	11.9	13.4	15.5	15.4	16.9
Constellation cost	0.429	0.507	0.449	0.503	0.552
	0.428	0.502	0.657	0.502	0.503
	0.427	0.504	0.452	0.502	0.503

## Data Availability

The data presented in this study are available on request from the corresponding author. The data are not publicly available due to private reasons.
